# Terminating B cell receptor signaling

**DOI:** 10.18632/oncotarget.22986

**Published:** 2017-12-06

**Authors:** Dara K. Mohammad, Beston F. Nore, C. I. Edvard Smith

**Affiliations:** Department of Laboratory Medicine, Clinical Research Center, Karolinska Institutet, Karolinska University Hospital Huddinge, Huddinge, Stockholm, Sweden

**Keywords:** BCR signaling, BTK, SYK, BLNK, AKT

Bruton's tyrosine kinase (BTK) mediated signaling is critical for B cell development and activation. The physiological role of BTK is evident since its functional loss results in a differentiation block of B cells beyond the pro-B cell stage causing X-linked agammaglobulinemia. Inhibitors of BTK, such as ibrutinib and acalabrutinib profoundly impair BCR signaling. They are highly effective in the treatment of many lymphoid tumors, including chronic lymphocytic leukemia, mantle cell lymphoma, Waldenström's macroglobulinemia and marginal zone lymphoma. Most patients respond, but escape mutants of different origins may appear [1–3]. The most common mutations affect BTK itself, impairing the binding of the inhibitors to cysteine 481 in the kinase domain. Others, instead, introduce changes in Phospholipase Cγ2 (PLCγ2), which is the major substrate of BTK, rendering this enzyme constitutively active and thereby signaling independently of BTK/BCR.

**Figure 1:**
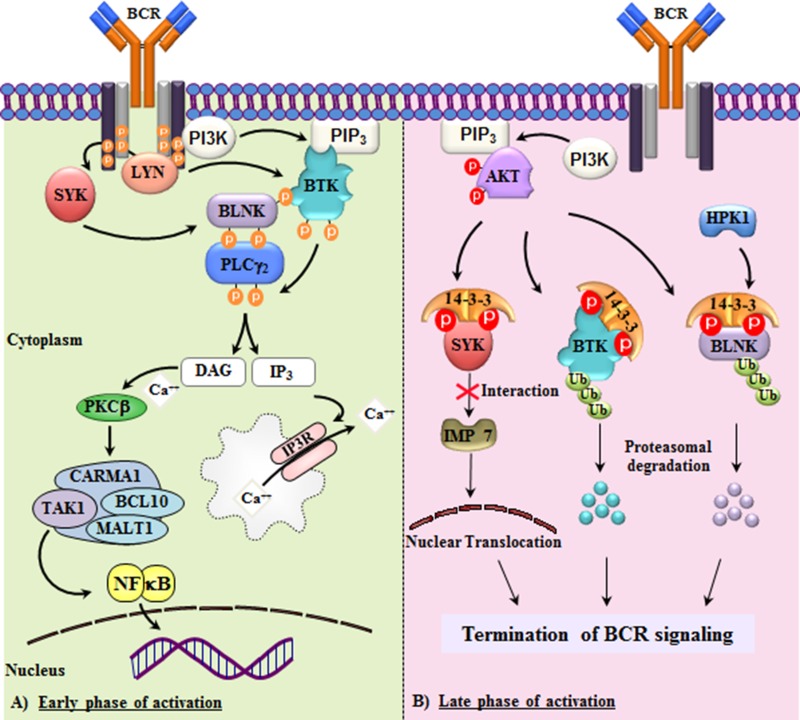
Termination of B-cell receptor (BCR) signaling by 14-3-3 through AKT and HPK1 **A.** BCR activation induces SYK, BTK and BLNK *tyrosine* phosphorylation and activation of downstream signaling through PLCγ2 and NF-κB [1]. **B.** Thereafter, BCR signaling is turned off: PI3-kinase mediates activation of AKT/PKB leading to *serine/threonine* phosphorylation of BTK (S51/T495) [5] and SYK (S295/S297) [7], while AKT and HPK1 jointly phosphorylate BLNK (S285 and T152, respectively) [7,8]. Subsequently, 14-3-3 proteins tether to the dual *serine/threonine* phosphorylated sites of target proteins. This promotes ubiquitination and degradation of activated BLNK and BTK, thereby attenuating BCR signaling. SYK kinase is affected differentially by inhibition of nuclear translocation. Tyrosine phosphorylations are in orange and serine/threonine phosphorylations in red. Red X indicates inhibition of SYK binding to Importin 7.

In light of the potent effect of BTK inhibitors, it seems important to consider the endogenous/physiological control of the BCR signaling pathway, including how activated BTK is turned off. Immediately upstream of BTK are SRC-family kinases (SFKs). They phosphorylate tyrosine 551 in BTK, which changes the conformation leading to an activated form of the kinase. In B lymphocytes LYN is the predominant member of this family (Fig. 1, panel A), while also BLK and FYN are expressed. SFKs are regulated in a unique way through the phosphorylation of a conserved C-terminal tyrosine. Upon this phosphorylation by the CSK kinase, the modified tyrosine tethers to the tyrosine-binding SH2 domain in *cis*, thereby impeding enzyme activity. This phosphorylation is reversible and the CD45 transmembrane phosphatase is directly involved [4].

Many tyrosine kinases, and not only SFKs, are themselves regulated by various forms reversible tyrosine phosphorylation mediated by other kinases and phosphatases. BTK regulation seems to differ, as up until today, there is no report demonstrating tyrosine dephosphorylation of BTK by phosphatases. Instead, it seems as if serine/threonine kinases (S/T kinases) are involved in the down-regulation of BCR signaling, often with 14-3-3 proteins being crucial contributors to this process. Examples of the BCR downstream proteins involved in this process are BTK, Spleen tyrosine kinase (SYK) and B cell linker/adapter protein (BLNK) [5]. Thus, we have previously shown that the S/T kinase, protein kinase B (AKT/PKB), phosphorylates two sites in BTK, one located in the Pleckstrin homology (PH) domain and the other in the kinase domain [5]. 14-3-3 proteins are small and acidic, lack enzymatic activity and naturally occur as dimers. Dual S/T phosphorylations make molecules ideal targets for this family of proteins. Binding of 14-3-3 proteins to BTK triggers proteasomal degradation (Fig. 1). Hence, following activation by the generation of pY551, the activated BTK molecules are selectively subjected to robust S/T phosphorylation and subsequently targeted for degradation.

In further support of the idea that S/T kinases down-regulate BCR signaling is the fact that Protein kinase C (PKC) β abates the activity of BTK by phosphorylating serine 180 in the Tec homology (TH) domain [6]. The underlying mechanism seems to impair membrane translocation and the subsequent phosphorylation of pY551, which is required for activation of BTK.

However, other molecules in the BCR signaling cascade are affected by S/T kinases too, such as the cytoplasmic linker/adaptor protein BLNK, also known as SLP-65. BLNK interacts with BTK and defects of either molecule cause related forms of primary immunodeficiency, both confined to the B-cell lineage. AKT is also implicated in the 14-3-3 mediated degradation of BLNK [7]. However, in this case another kinase, HPK1 [8] is involved too, and together with AKT generate the dual S/T phosphorylation.

Finally, the SYK kinase, which is another key player for BCR signaling initiation, is also affected by AKT phosphorylation (Fig. 1). However, the consequence of this post-translational modification is less clear, but seems to involve SYK's interaction with BLNK as well as its nuclear translocation [8]. In this context it is interesting to note that BTK also shuttles between the cytoplasm and the nucleus, although the biological ramifications of this phenomenon remain elusive. Collectively these findings support a scheme, where BCR signaling is turned on by tyrosine kinases and off by S/T kinases. This may have implications for future pharmaceutical interventions other than inhibitors of BTK's catalytic activity.
